# A strong and ductile medium-entropy alloy resists hydrogen embrittlement and corrosion

**DOI:** 10.1038/s41467-020-16791-8

**Published:** 2020-06-17

**Authors:** Hong Luo, Seok Su Sohn, Wenjun Lu, Linlin Li, Xiaogang Li, Chandrahaasan K. Soundararajan, Waldemar Krieger, Zhiming Li, Dierk Raabe

**Affiliations:** 1 Institute for Advanced Materials and Technology, University of Science and Technology Beijing, Beijing, 100083 China; 2 Max-Planck-Institut für Eisenforschung, Max-Planck-Straße 1, 40237 Düsseldorf, Germany; 3 Department of Materials Science and Engineering, Korea University, Seoul, 02841 South Korea; 4 School of Materials Science and Engineering, Central South University, Changsha, 410083 China; 5 State Key Laboratory of Powder Metallurgy, Central South University, Changsha, 410083 China

**Keywords:** Mechanical engineering, Metals and alloys

## Abstract

Strong and ductile materials that have high resistance to corrosion and hydrogen embrittlement are rare and yet essential for realizing safety-critical energy infrastructures, hydrogen-based industries, and transportation solutions. Here we report how we reconcile these constraints in the form of a strong and ductile CoNiV medium-entropy alloy with face-centered cubic structure. It shows high resistance to hydrogen embrittlement at ambient temperature at a strain rate of 10^−4^ s^−1^, due to its low hydrogen diffusivity and the deformation twinning that impedes crack propagation. Moreover, a dense oxide film formed on the alloy’s surface reduces the hydrogen uptake rate, and provides high corrosion resistance in dilute sulfuric acid with a corrosion current density below 7 μA cm^−2^. The combination of load carrying capacity and resistance to harsh environmental conditions may qualify this multi-component alloy as a potential candidate material for sustainable and safe infrastructures and devices.

## Abstract (web summary)

Strong and ductile materials with resistance to both corrosion and hydrogen embrittlement remain rare and yet are essential for hydrogen-propelled industries. Here, the authors show that a CoNiV medium-entropy alloy with face-centered cubic structure fulfils all the above criteria.

## Introduction

Machines, infrastructures, power plants, and constructions that surround us depend critically on materials with high strength and damage tolerance^1,2^. However, these mechanical properties gradually or even catastrophically deteriorate under the influence of hydrogen, acidic or mixed environments, due to hydrogen embrittlement (HE)^3–8^ and corrosion^9–11^. Some of the most advanced commercial high-strength alloys available today such as stainless steels, nickel-based superalloys, titanium alloys, and aluminum alloys^12–18^ have reached in part good resistance to corrosion and—to a lesser extent—to hydrogen embrittlement through composition adjustment, microstructure, and coating^19–22^. However, when enhancing the alloys’ strength levels further, a fundamental problem is encountered between measures that improve mechanical strength and those that improve resistance to environmental attack. The reason is that those microstructure modifications that increase strength the most, e.g., interfaces and second phase precipitates create local differences in electrochemical potential, which leads to galvanic corrosion. Similarly, hydrogen gets accumulated at interfaces, inside precipitates and in regions of high micromechanical contrast, causing hydrogen-enhanced plastic softening or decohesion. Therefore, it is important to explore concepts for alloys that reconcile mechanical properties in the presence of hydrogen with high resistance against acidic environmental attack. High- and medium-entropy alloys (HEAs & MEAs) represent a metallurgical concept that shifts compositions from the corners of multi-component alloy phase diagrams toward their centers^23,24^. MEAs were defined as alloys with a mixing entropy between 1*R* and 1.5*R*, where *R* is the gas constant^25^. These materials may offer avenues for good mechanical performance and high environmental stability.

In this work, we present an equiatomic CoNiV MEA, which maintains its good mechanical performance under the impact of hydrogen, i.e., the material under investigation shows no obvious hydrogen embrittlement at a temperature of 300 K and a strain rate of 10^−4^ s^−1^, and it has good corrosion resistance when exposed to acidic environments. This property synergy, balancing strength, ductility, corrosion, and hydrogen embrittlement, is realized by four main effects: The alloy’s solid solution hardened single face-centered cubic (f.c.c.) phase structure, devoid of any precipitations, provides both, (1) low hydrogen diffusivity and (2) absence of local electrochemical potential gradients. (3) The solute hydrogen in the bulk alloy matrix leads to the formation of deformation twins which impede crack propagation during slow strain rate tensile testing^26,27^. (4) The formation of an oxide barrier film with low concentrations of point defects, i.e., metal and oxygen vacancies and metal interstitials, and low content of Co- and Ni-containing hydroxides on the alloy surface in sulfuric acid solution acts as an efficient barrier against oxygen transport, thereby enhancing corrosion resistance. In addition, the surface oxide film can further reduce the hydrogen embrittlement by lowering the rate of hydrogen uptake on the surface.

## Results

### Processing and microstructure

**Fig. 1:**
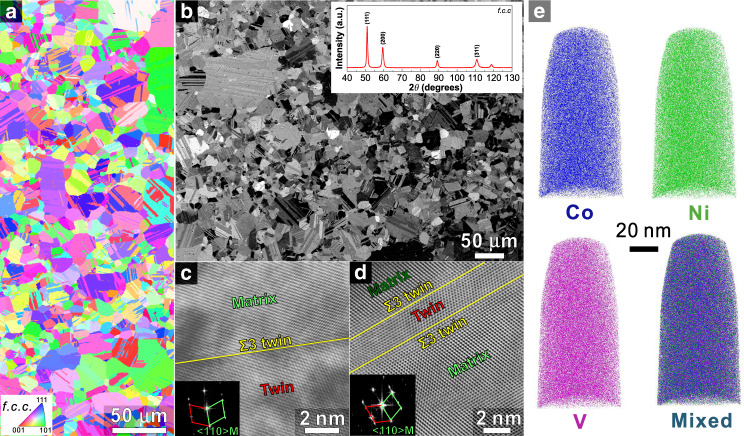
Recrystallization microstructures of the equiatomic CoNiV MEA. **a** IPF map. **b** ECC image with inserted XRD pattern. **c**, **d** High angle annular dark-field (HAADF) images of twins and the corresponding selected area diffraction (SAD) patterns. **e** 3D reconstructions of a typical APT tip. XRD X-ray diffraction, IPF inverse pole figure, ECCI electron channeling contrast imaging, APT atom probe tomography.

We prepared the equiatomic CoNiV MEA by vacuum induction melting and drop-casting using pure metals. Subsequently, the ingots were homogenized at 1200 °C for 24 h under Ar atmosphere. To refine the grain size, the plates were cold-rolled to 75% thickness reduction and then recrystallization annealed at 950 °C for 1 h in the Ar atmosphere. The chemical composition of the MEA is given in the Supplementary Table 1. Figure 1 shows the recrystallized microstructure and elemental distribution in the CoNiV MEA. The material has an average grain size of ~10.5 μm (annealing-twin boundaries are all included) with a random crystallographic texture (Fig. 1a). The grain size distribution is a bit inhomogeneous, which is related to the processing conditions. Some randomly distributed annealing twins are observed in the microstructure. The X-ray diffraction (XRD) pattern inserted in Fig. 1b confirms the single f.c.c. phase structure, consistent with Thermo-Calc calculations (TCHEA3 database (Supplementary Fig. 1)). Electron channeling contrast imaging (ECCI) (Fig. 1b), transmission electron microscopy (TEM) and the corresponding fast Fourier transform images (Fig. 1c, d) confirm the existence of annealing twins in the microstructure. Tips for atom probe tomography (APT) to reveal the nanoscale elemental distribution. The results show that no apparent local chemical ordering is observed in the 3D reconstructions (Fig. 1e), indicating that the present alloy is in a solid solution state.

### Resistance to hydrogen embrittlement

**Fig. 2:**
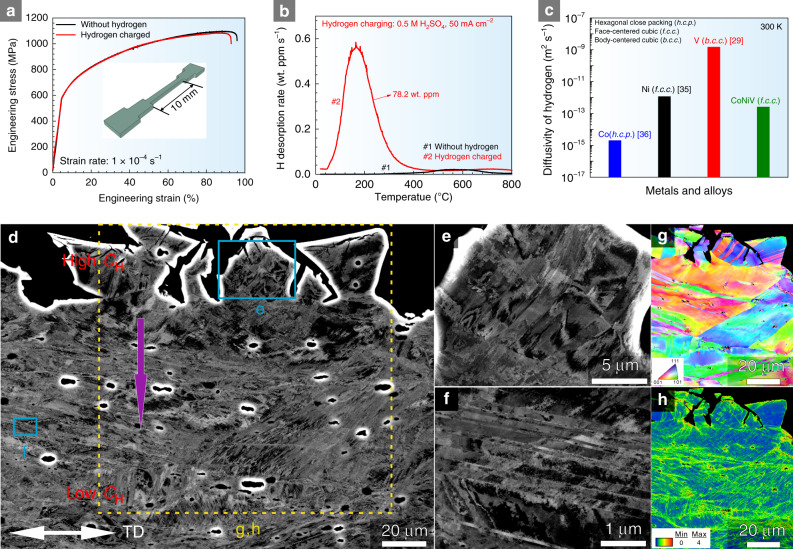
Mechanical behavior and deformation microstructures near the fracture surfaces of equiatomic CoNiV MEA. **a** Mechanical behavior with/without in situ hydrogen charging, the samples were charged in 0.5 M H_2_SO_4_ solution with 5 g L^−1^ thiourea at a current density of 50 mA cm^−2^. The inserted figure shows the used tensile sample geometry. **b** Typical hydrogen desorption rate curve of the hydrogen-charged and tensile-tested samples. Prior to the in situ tensile testing, the samples were pre-charged for 24 h at 50 mA cm^−2^. **c** Hydrogen diffusivity of equiatomic CoNiV MEA at 300 K. The hydrogen diffusivity values of pure Ni^35^, Co^36^, and V^29^ metals were selected as reference. **d**–**h** ECC images of deformation microstructures near the fracture surfaces of MEA samples charged with hydrogen. *C*_H_ and TD refer to the hydrogen concentration and tensile direction, respectively.

Figure 2a shows the slow strain rate (1 × 10^−4^ s^−1^) tensile deformation behavior of the CoNiV MEA with and without in situ hydrogen charging at a temperature of 300 K. The data stem from three tensile experiments conducted for each material state. The CoNiV MEA without hydrogen shows an ultimate tensile strength (UTS) of 1095 ± 10 MPa and a total elongation of 85.8 ± 2.0%, promoted by the high lattice friction enabled by the massive solid solution, dislocation-mediated plasticity^28^, and the high strain hardening. The UTS and total elongation values of the hydrogen-charged samples are only marginally reduced to approximately 1079 ± 12 MPa and 84.3 ± 1.5%, respectively. Figure 2b shows the hydrogen desorption rate curves for the in situ hydrogen-charged MEA samples. Although the distribution of hydrogen inside the sample is expected to be inhomogeneous due to the electrochemical hydrogen charging protocol, the total hydrogen concentration can be calculated through analysis of the TDS curves, and is ~78.2 wt ppm. In addition, the hydrogen diffusion coefficient in the bulk of the CoNiV MEA is ~10^−13^ m^2^ s^−1^, which is much lower than that of hydrogen in pure V (~10^−9^ m^2^ s^−1^)^29^ (Fig. 2c). The low total hydrogen diffusivity in the current MEA is related to two essential aspects, associated with the massive addition of Ni and Co to the matrix: the first one is the formation of a single f.c.c. solid solution, which generally exhibits low hydrogen diffusivity. The second one is the formation of a dense surface barrier film, impeding the rate of hydrogen liberation on the surface.

To reveal the micro-mechanisms behind the resistance of this MEA to hydrogen embrittlement at a temperature of 300 K and a strain rate of 10^−4^ s^−1^ compared for instance to that of nickel-based alloys^30^, we probed the deformation microstructures in the cross-sections of the tensile-tested CoNiV MEA samples that had been in situ exposed to hydrogen charging. Figure 2d, f shows ECC images near the fracture surfaces of the material charged with hydrogen. The surface regions of the samples have relatively high concentration of hydrogen. This causes the formation of microcracks during the plastic deformation at medium strain levels. Although V and Ni have been discussed as being potentially susceptible to hydride formation during intense hydrogen charging, no evidence of hydrides is found in this MEA, even after 60 days of electrochemical hydrogen charging. The absence of hydrides after hydrogen charging was confirmed by XRD (Supplementary Fig. 2) and high-resolution TEM analysis (Supplementary Fig. 3). Also, the ECC images (Fig. 2e, f) show different deformation mechanisms in the presence of hydrogen, such as deformation-induced cracking near the surfaces, due to the high hydrogen concentrations in these regions, and deformation-induced nanotwins, which were also observed in CoCrFeMnNi high-entropy alloys^31^, Fe–Ti–C alloys^32^ and Fe–30Mn–Si–Al austenitic alloys^33^ under enhanced hydrogen concentrations. In principle, the flow stress could be slightly enhanced due to the hydrogen-related formation of nanotwins. However, the relatively high concentration of hydrogen in the surface regions leads also to surface microcracks, an effect which in turn can slightly deteriorate the material’s flow stress, due to the associated microscopic notches. The positive influence of the nanotwins and the negative effect of the near-surface microcracks compete so that the flow stress and strain hardening values remain weakly affected by hydrogen charging, i.e., no substantial changes in flow stress are observed in the tensile curves when probed under hydrogen.

The kernel average misorientation (KAM) map in Fig. 2h shows that the hydrogen-induced cracks are surrounded by zones of high local strains. Hydrogen-induced cracking is controlled by diffusion of hydrogen and its accumulation at crack tips where hydrogen transport can be enhanced by dislocations. Moreover, it also shows several sharp peaks, which translate to high densities of geometrically necessary dislocations. These regions create high local dislocation pile-up stresses which further promote the accumulation of hydrogen which in turn fuels the hydrogen enhanced local plasticity effect^34^.

Among the different deformation mechanisms encountered in this material, the stacking faults energy (SFE) plays a particularly important role in the strain hardening. It affects the splitting of lattice dislocations into partials, which reduces cross-slip and leads to sessile dislocation reaction products. Hence, it is a key parameter that governs strain hardening^37^. In the current alloy charged with hydrogen, we find mechanical twins but no martensite, similar to that in the CoCrFeMnNi HEA^38^. The positive effects of twinning on strain hardening are due to the dynamic Hall–Petch effect^39^, i.e., the twin interfaces reduce the dislocations’ mean free path, so that the dislocation flux can only be replenished by producing fresh dislocations at the cost of higher stresses^40,41^. The beneficial effect of twinning on the material’s resistance to hydrogen embrittlement was also observed and demonstrated in other f.c.c. alloys, e.g., Fe–30Mn–Si–Al austenitic alloys^33^ and single-crystalline Hadfield steel^42^. On the other hand, twin boundaries, especially the coherent ones, are inherently more resistant to hydrogen embrittlement than less ordered interfaces because of their high surface separation energy and low hydrogen solubility^21,43^. We also find that crack propagation is impeded when a crack encounters twin boundaries (Supplementary Fig. 4).

**Fig. 3:**
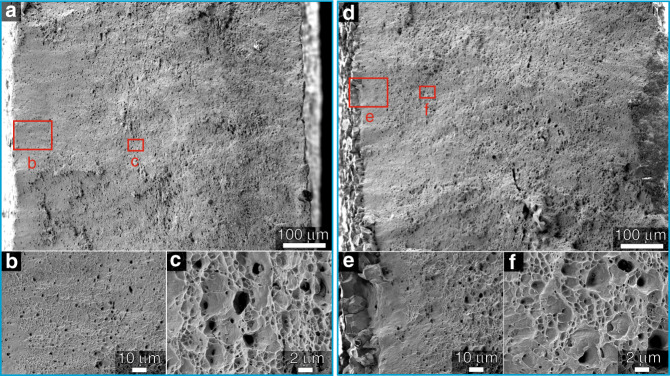
Fracture surfaces of hydrogen pre-charged and uncharged equiatomic CoNiV MEA after tensile test. **a** Fracture morphologies of MEA samples after tensile testing without hydrogen charging, showing ductile dimple fracture surface. **b**, **c** Enlarged images for edge and interior regions of the fracture surface marked in (**a**), respectively. **d** Hydrogen pre-charged sample showing both brittle and ductile fracture features. **e**, **f** Enlarged images for surface and interior regions of the fracture surface marked in (**d**), respectively.

To better understand the fracture behavior of the CoNiV MEA in the presence of hydrogen, we investigated the morphology of the fracture surfaces of all samples after in situ tensile testing. Figure 3a shows a fully ductile fracture, characterized by the growth and coalescence of microvoids in both, the edge and center regions of the sample devoid of hydrogen. Some particles, which had acted as typical initiation sites of microvoids, were found inside the voids on the fracture surface (Fig. 3c). In contrast, a mixed failure mode was observed in the hydrogen-charged CoNiV MEAs: the interior regions of the sample exhibit microvoid coalescence and ductile fracture. Precipitations^44^ and hydrides^45^ were reported to promote hydrogen embrittlement by initializing micro-cracks/voids or decohesion effects from precipitations/matrix interfaces. Therefore, alloys with solid solution single f.c.c. structure, which are devoid of precipitates, e.g., such as the current CoNiV MEAs, exhibit higher resistance to hydrogen embrittlement than materials that contain precipitates. However, because of the high hydrogen concentration accumulated during electrochemical cathodic charging, the surface edges of the current material show intergranular fracture. The length of the brittle zone was below 20 μm, further confirming the low hydrogen diffusivity value (~10^−13^ m^2^ s^−1^) in this MEA.

### Corrosion behavior

**Fig. 4:**
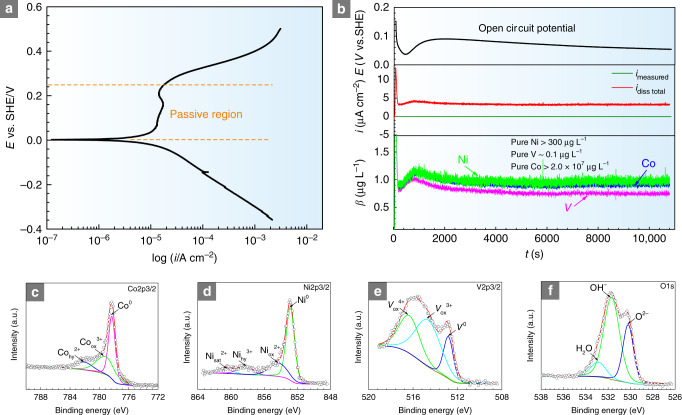
Electrochemical behavior and the composition of the surface barrier film. **a** Potentiodynamic polarization curve of MEA in 0.1 M H_2_SO_4_ solution. **b** Online ICP-MS dissolution profiles were converted into dissolution concentration values of metallic-Co, Ni, and V shown in blue, green, and pink color codes, respectively. For comparison, we also measured the dissolved concentrations of Co, Ni, and V ions from their respective pure metals under the same conditions. **c**–**f** XPS spectra of Co2*p*3/2, Ni2*p*3/2, V2*p*3/2, and O1*s* of the barrier films formed on equiatomic CoNiV MEA in 0.1 M H_2_SO_4_ solution.

Figure 4a shows the potentiodynamic polarization curve of CoNiV MEA in 0.1 M H_2_SO_4_ solution, indicating the formation of a stable barrier film during electrochemical corrosion. It should be noted that when the final scanning potential exceeds any potentials in the passive region of the MEA, formation of a scaly surface morphology can be observed after potentiodynamic polarization curve testing (Supplementary Fig. 5), indicating the occurrence of general corrosion in 0.1 M H_2_SO_4_ solution under a high applied potential, which is similar to that observed in other corrosion resistant alloys^46^. The high corrosion resistance of the MEA was further validated by element-resolved in situ analysis in 0.1 M H_2_SO_4_ (Fig. 4b) under open-circuit potential (OCP) conditions. The results show that dissolved concentrations of Co and Ni ions (~0.9, ~1.1 μg L^−1^) from the current MEA are much lower than the concentrations of the same ions from their respective pure metals (~2 × 10^7^, ~300 μg L^−1^). For comparison, we also measured the dissolved concentrations of Co, Ni, and V ions from their respective pure metals under the same conditions. However, compared with the dissolved concentration of V ions (~0.1 μg L^−1^) from pure V, the concentration of V ions (~0.8 μg L^−1^) dissolving from the current MEA is slightly higher. These findings indicate that the barrier film formed on the current CoNiV MEA can prevent the dissolution of Ni, Co metals from the substrate. On the other hand, the features reveal that the addition of V increases the stability of the barrier film in CoNiV^47,48^, which is also confirmed by Mott–Schottky measurements (Supplementary Fig. 6). Compared with the Mott–Schottky plots of pure Co, Ni, and V in 0.1 M H_2_SO_4_ solution, the current MEA displays very similar semiconductor features, indicating *n*-type and *p*-type semiconductor responses. The level of point defects in this MEA is calculated according to Eq. (1) as listed in Methods. As estimated from the values from the linear part of the slopes of the Mott–Schottky plots, the doping density, which indicates the stability of barrier film of the current MEA is lower than that of pure Co and Ni, but slightly higher than that of pure V.

X-ray photoelectron spectroscopy (XPS) was used to further determine the surface composition of the equiatomic CoNiV MEA after exposure to 0.1 M H_2_SO_4_ solution. Figure 4c, f shows the elements peak fitting results of Co2*p*_3/2_, Ni2*p*_3/2_, V2*p*_3/2_, and O1*s* in the barrier film for the MEA after passivation 0.5 h at +400 mV_SHE_ in 0.1 M H_2_SO_4_ solution (for comparison, the detailed surface compositions of each pure metal are also listed in the Supplementary Fig. 7). It is observed that the surface layer of the MEA consists predominantly of Co-, Ni-, and V-containing oxides and hydroxides. The V and Ni oxides in the MEA show beneficial effects to the corrosion resistance in acidic solution. It is pertinent to note that—compared with the compositions of the barrier film formed on pure metals—the concentrations of Ni- and Co-hydroxide in the MEA are significantly lower (see the Supplementary Table 2), i.e., the content of Co-hydroxide decreases from 28.1 at.% (in pure metal) to 2.9 at.% (in MEA) and the content of Ni-hydroxide decreases from 13.6 at.% (in pure metal) to 2.5 at.% (in MEA). High concentrations of metallic hydroxides in the film have been found before to act in a detrimental manner on the corrosion resistance^49^.

## Discussion

**Fig. 5:**
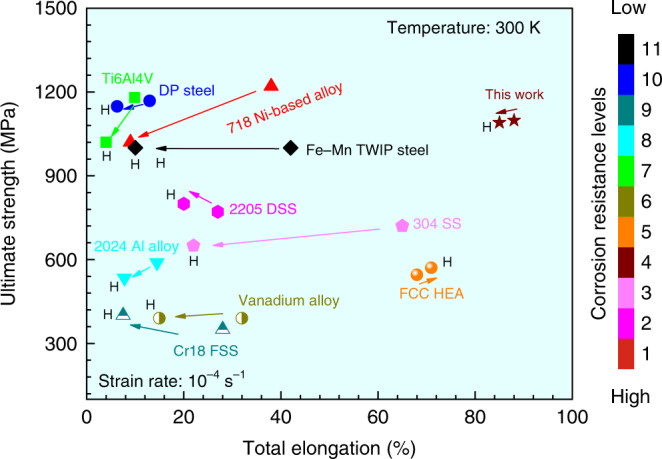
A summary of total elongation vs. ultimate strength for various alloys with/without hydrogen. The diagram compiles our current results on the equiatomic CoNiV MEA, TWIP steel^50^, high-strength dual-phase steel (DP)^51^, austenitic stainless steel (SS), ferritic stainless steel (FSS)^52,53^, duplex stainless steel (DSS)^54,55^, nickel-based alloy^30,56^, vanadium alloy^57^, aluminum alloy^58^, titanium alloy^59^, and Cantor alloy (f.c.c. HEA)^31^. All samples were probed at the same slow strain rate level (10^−4^ s^−1^) and at the same temperature of 300 K. The combination of high resistance to hydrogen embrittlement and corrosion together with its maintained mechanical properties observed in the equiatomic CoNiV MEA exceeds that of many other alloys. The corrosion resistance levels shown here have been calculated using the corrosion current density in 0.1 M H_2_SO_4_ solution, changing from high (1) to low (11) as expressed by the color legend. The arrows in this Ashby diagram show in which direction the mechanical properties change when the alloys are exposed to hydrogen. Another Ashby plot compiling yield strength, elongation, resistance to hydrogen embrittlement and corrosion is shown in the Supplementary Fig. 8.

With respect to the alloy’s resistance to hydrogen embrittlement and corrosion, a comparison with the other major material classes is shown on the Ashby plot compiling ultimate strength, elongation, resistance to hydrogen embrittlement and corrosion (Fig. 5). Another Ashby plot compiling yield strength, elongation, resistance to hydrogen embrittlement and corrosion is shown in the Supplementary Fig. 8. The yield strength of the CoNiV MEA is ~600 MPa and its ultimate strength is ~1 GPa. The comparison thus reveals that the CoNiV MEA shows a good strength–ductility relation combined with high resistance to corrosion. Moreover, the MEA exhibits high resistance to hydrogen embrittlement at a temperature of 300 K and a strain rate level of 10^−4^ s^−1^.

We show here that the equiatomic CoNiV MEA has not only a high ultimate tensile strength of 1 GPa at >90% elongation but it has also exceptional resistance to corrosion and hydrogen embrittlement at a temperature of 300 K at a strain rate of 10^−4^ s^−1^. More specific, the total elongation drops by only ~1.5% in the presence of hydrogen, and the corrosion current density remains below 7 μA cm^−2^ (see the Supplementary Table 3 and Fig. 5). We also reveal the mechanisms behind the combined balancing of such opposing constraints as strength, ductility, corrosion, and hydrogen embrittlement in this material. The main effects are that (i) single phase solid solution hardened single f.c.c. structures, devoid of precipitates, create only modest internal micromechanical contrast which suppresses high local hydrogen accumulation; (ii) the stacking fault energy of the alloy is in a range where it gets slightly reduced by hydrogen, promoting formation of nanotwins and enhanced local strain hardening which impedes crack propagation; (iii) the used solid solution elements lead to a low hydrogen diffusion coefficient and do not promote formation of hydrides (which are otherwise typical crack initiation sites); (iv) the alloy forms a stable oxide film with a low concentration of point defects and metal hydroxides which inhibits hydrogen liberation and diffusion as well as corrosion.

Our work thus opens pathways to designing alloys with a good mechanical performance at high environmental stability. It also shows that strategies to reconcile conflicting features such as micromechanical strengthening on the one hand and environmental attack on the other hand require a holistic design approach based on a solid understanding of the underlying strengthening and weakening mechanisms.

## Methods

### Materials preparation

The equiatomic CoNiV MEA ingot was prepared in a vacuum induction chamber of the furnace protected under argon atmosphere by melting the pure metallic ingredients with the nominal composition Co_33.4_Ni_33.3_V_33.3_ (at.%). The dimension of the cast and as-prepared ingot was 100 × 35 × 8 mm^3^. To chemically homogenize the as-cast alloy, the ingot was subsequently subjected to a heat-treatment at a temperature of 1200 °C for 24 h in evacuated quartz ampules, then water-quenching was conducted. A 20 wt% HCl solution was used to remove the surface oxide scales formed during the heating process. Thereafter, the average grain size of the samples was refined by a multi-step cold-rolling procedure to a final total thickness reduction of 75% and subsequent recrystallization annealing under protective argon atmosphere at a furnace temperature of 950 °C for 1 h. After that, the samples were water-quenched.

### Microstructural and elemental characterization

An X-ray equipment ISO-DEBYEFLEX 3003, operating with a short anodic tube source producing Co Kα radiation (*λ* = 1.788965 Å, 40 kV, and 30 mA), were used to carry out the X-ray diffraction (XRD) measurements. Electron backscatter diffraction (EBSD) measurements were performed in a Zeiss-Crossbeam XB 1540 FIB scanning electron microscope. The characterization of the deformation microstructure by electron channeling contrast imaging (ECCI) and the analysis of the fracture morphology were performed using a Zeiss-Merlin high-resolution field emission electron microscope. The atom probe tomography (APT) tips were produced via a focussed ion beam (FIB, FEI Helios Nanolab 600i) from suited regions of interest containing grain boundaries that were beforehand revealed by a preceding EBSD scan. The elemental distributions of the recrystallized alloy were investigated using APT (LEAP 3000X HR, Cameca Inc.) operated under voltage-pulsed mode. TEM foil samples were prepared by a FIB lift-out procedure, imposing a final cleaning voltage of 5 kV. HAADF scanning transmission electron microscopy (STEM) imaging was conducted in an aberration-corrected FEI Titan Themis. The acceleration voltage was 300 kV. A probe semi-convergence angle of 17 mrad with inner and outer semi-collection angles of the annular detector from 73 to 350 mrad were selected to obtain the high-resolution imaging.

### Hydrogen charging and mechanical characterization

Hydrogen charging was carried out by an electrochemical method at 50 mA cm^−2^ current density in a 0.5 M H_2_SO_4_ solution with 5 g L^−1^ thiourea (CH_4_N_2_S) at the temperature of 298 K. The counter electrode was a piece of platinum plate. The samples were first pre-charged for 24 h at 50 mA cm^−2^. After that they were continuously in situ charged during the entire duration of the tensile tests. The uniaxial tensile tests were conducted in an Instron tensile machine with an initial strain rate of 1 × 10^−4^ s^−1^ at a temperature of 300 K. The tensile specimens with gage dimensions of 3 mm × 1 mm × 10 mm were prepared along the rolling direction by spark cut. The gage part of the tensile specimens was charged with hydrogen. The hydrogen desorption was investigated via a custom-designed UHV-based Thermal Desorption Analysis instrument in conjunction with a Mass Spectrometer detector. The heating temperature range was selected from 25 °C up to 800 °C with a constant heating rate of 26 °C min^−1^. The total hydrogen concentration stored in the MEA samples was determined by conducting cumulative desorbed hydrogen probing during a heating experiment using a gradual heating rate up to 800 °C.

### Hydrogen permeation test

The hydrogen permeation tests were carried out in a Devanathan–Stachurski cell with a hydrogen charging cell (the solution was 0.5 M H_2_SO_4_) and a hydrogen oxidation cell (the solution was 0.1 M NaOH) at 300 K with a disk sample of 10 mm in diameter and a thickness of 0.15 mm. The disk samples were ground using SiC papers from 400 to 2000 grits followed by final polishing with 0.3 μm diamond powders. After cleaning, one side of the samples were plated with Pd (~100 nm in thickness). In the charging part, hydrogen charging was performed at a constant current density of 50 mA cm^−2^. In the oxidation part, a constant potential of +200 mV (vs. Ag/AgCl) was applied on the oxidation cell and hydrogen penetration into the disk membrane by diffusion was measured by the oxidation current density during the whole process through a Solartron 1287 potentiostat. Prior to hydrogen generation, the background current density in the oxidation part was below 50 nA cm^−2^, ensuring that all measured anodic current densities are induced by hydrogen. The electrolytes were purged with high purity argon during the whole experimental process.

### Electrochemical measurements and online in situ element-resolved analysis

Online element-resolved measurements were carried out in 0.1 M H_2_SO_4_ via a scanning flow cell (SFC) connected to the inductively coupled plasma-mass spectrometer (ICP-MS, NexION 300X, Perkin Elmer) with a potentiostat (Gamry Reference 600) at 298 K. A Pt-wire was placed in the inlet channel, serving as the counter electrode and the Ag/AgCl (3 M KCl) in the outlet channel of the microcell as the reference electrode. The tip of the microcell was pressed onto the sample, which acted as a work electrode. Between the tip and the sample, a silicone O-ring was encircled to prevent the leakage of solution during the measurements. The electrochemical and corrosion tests were conducted with a conventional three-electrode cell system in 0.1 M H_2_SO_4_ by Solartron system-potentiostat (model 1287 A)/frequency response analyzer (model 1260A). A saturated calomel electrode (SCE) and a piece of platinum sheet were selected as the reference and counter electrodes, respectively. The MEA sample was used as the work electrode. Prior to the electrochemical measurements, the open-circuit potential of the sample in the solution was measured for 30 min until reaching a stable status. Potentiodynamic polarization curves were started from the cathodic potential to the anodic potential corresponding to a current density value of 1 mA cm^−2^ at a scanning rate of 0.5 mV s^−1^. Mott–Schottky plots were constructed in the potential range from −1000 to 1000 mV_SCE_ with an appropriate frequency of 1 kHz. The scanning rate was 50 mV per step to avoid any changes in the defect structure of the barrier film. The concentrations of point defects in the barrier film were related to the linear portion of the slopes of the plots. They could be calculated by the following Eq. (1):

1 $$\frac{1}{{C^2}} = \frac{{ - 2}}{{e \cdot N_{\mathrm{q}} \cdot \varepsilon \cdot \varepsilon _0A^2}}\left( {E - E_{{\mathrm{FB}}} - \frac{{kT}}{e}} \right),$$

where *A* is the area, *ε*_0_ is the vacuum permittivity, *k* is the Boltzmann’s constant, *ε* is the relative dielectric constant, *e* is the electron charge, *N*_q_ is the concentrations of point defects, *T* is the absolute temperature, *E*_FB_ is the flat band potential, and *E* is the applied electrode potential.

### Compositional analysis of the barrier film

A Physical Electronics Quantum 2000 ESCA microprobe system was used to character the compositions of the barrier films of the MEA. The monochromatic Al Kα was selected as the X-ray source. The spectra were fitted by using a mixture of Gaussian–Lorentzian functions on a Shirley background via the software CasaXPS (version 2.3.15). The binding energy scale was calibrated with respect to the C1*s* peak at 285 eV, as a standard peak.

## Supplementary information

Supplementary Information

## Data Availability

The datasets generated during and/or analyzed during the current study are available from the corresponding author on reasonable request.
