# Author Correction: A strong and ductile medium-entropy alloy resists hydrogen embrittlement and corrosion

**DOI:** 10.1038/s41467-020-17295-1

**Published:** 2020-07-02

**Authors:** Hong Luo, Seok Su Sohn, Wenjun Lu, Linlin Li, Xiaogang Li, Chandrahaasan K. Soundararajan, Waldemar Krieger, Zhiming Li, Dierk Raabe

**Affiliations:** 1 Institute for Advanced Materials and Technology, University of Science and Technology Beijing, Beijing, 100083 China; 2 Max-Planck-Institut für Eisenforschung, Max-Planck-Straße 1, 40237 Düsseldorf, Germany; 3 Department of Materials Science and Engineering, Korea University, Seoul, 02841 South Korea; 4 School of Materials Science and Engineering, Central South University, Changsha, 410083 China; 5 State Key Laboratory of Powder Metallurgy, Central South University, Changsha, 410083 China

**Keywords:** Mechanical engineering, Metals and alloys

Correction to: *Nature Communications* 10.1038/s41467-020-16791-8, published online 17 June 2020.

The original version of this Article contained an error in the author affiliations.

The affiliation of Hong Luo and Xiaogang Li with Beijing Advanced Innovation Center for Materials Genome Engineering, Beijing 100083, China was inadvertently omitted.

This has now been corrected in both the PDF and HTML versions of the Article.

