# Supplementary material for: A strong and ductile medium-entropy alloy resists hydrogen embrittlement and corrosion
Source: Nat Commun. 2020 Jun 17;11:3081. doi: 10.1038/s41467-020-16791-8 (PMC7299985; doi:10.1038/s41467-020-16791-8)
Supplement: Supplementary file 1 — Supplementary Information [file 41467_2020_16791_MOESM1_ESM.pdf]

Supplementary information

A strong and ductile medium-entropy alloy resists hydrogen embrittlement and corrosion

Luo et al.

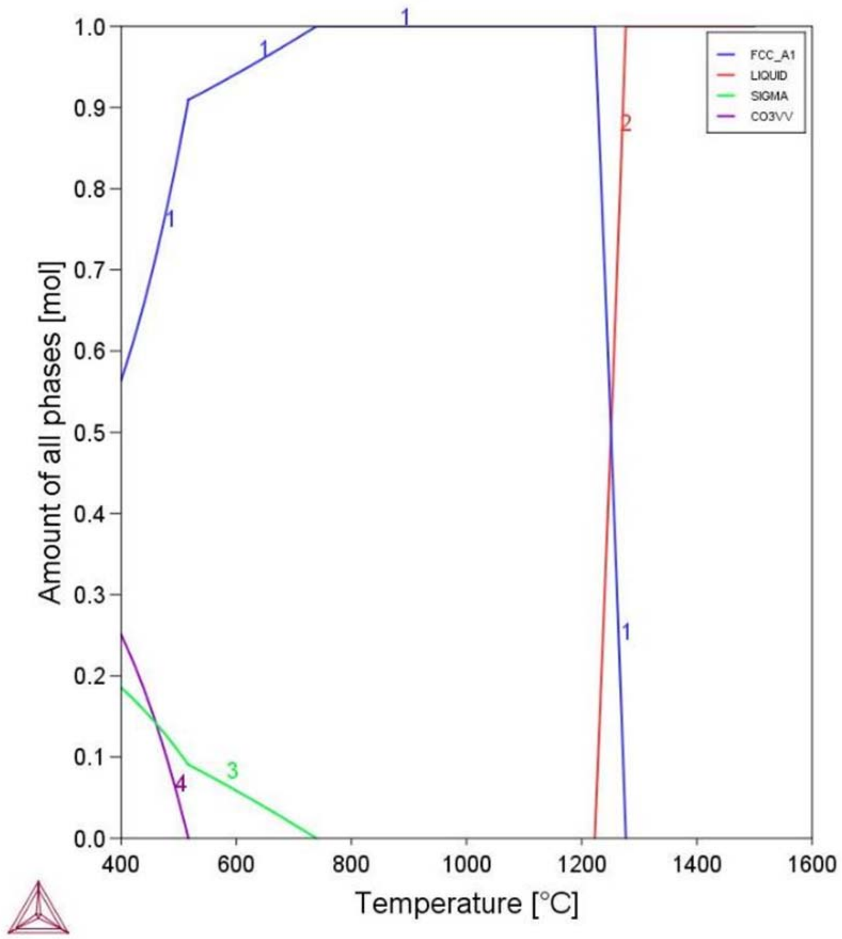

Supplementary Figure 1 | Calculated molar fraction of all equilibrium phases for various temperatures of the sample by Thermo-Calc in conjunction with the TCHEA3 database.

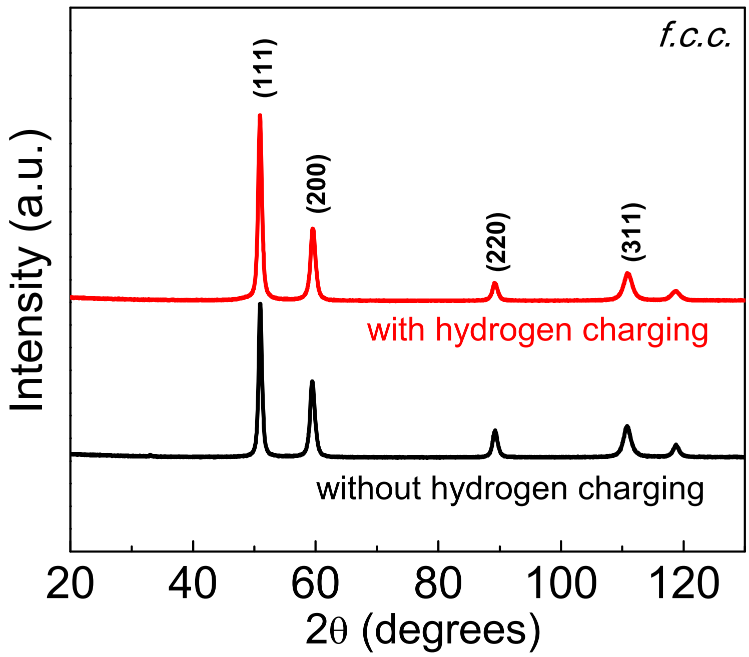

Supplementary Figure 2 | XRD analysis of the sample without and with hydrogen charging. The sample was hydrogen charged for 60 days at 25 mA cm^{-2} in 0.1 M H_2SO_4 solution.

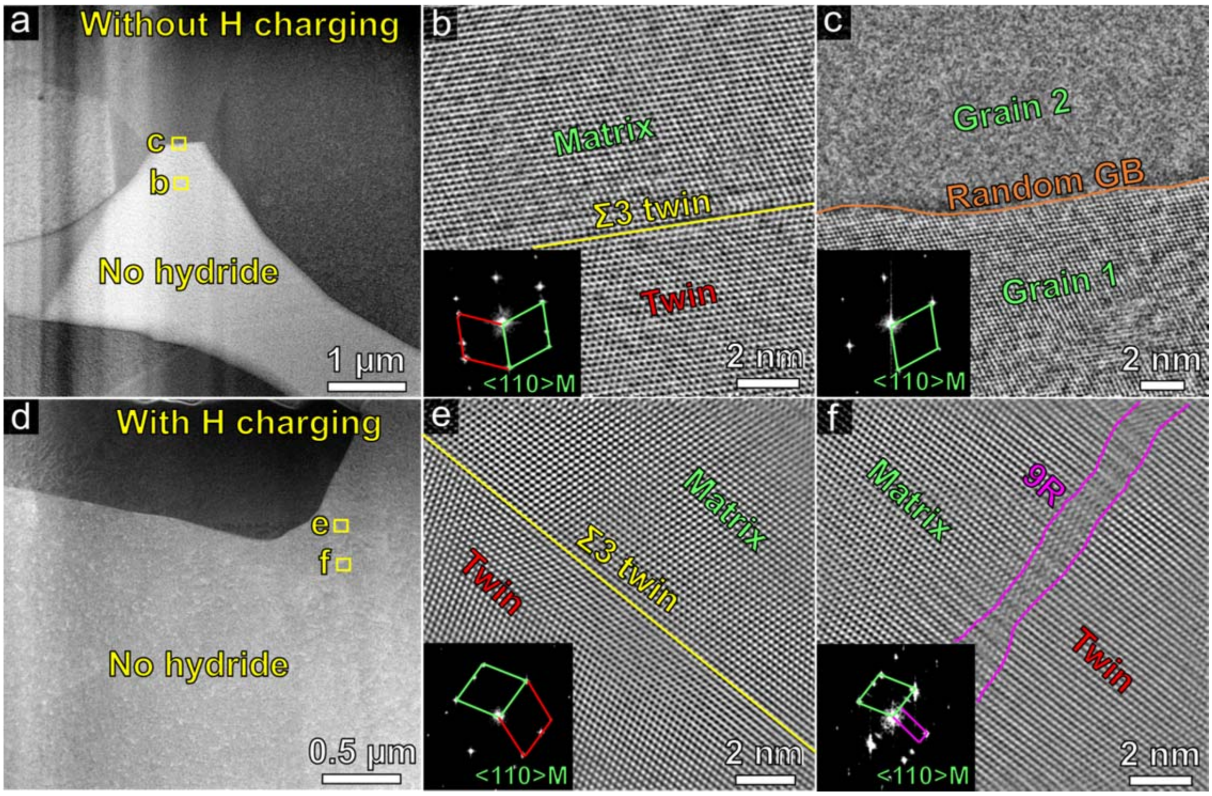

Supplementary Figure 3 | TEM/STEM analysis of the sample without and with hydrogen charging. a~c, Sample without hydrogen charging. **d~f**, Sample charged for 60 days at 25 mA cm^{-2} in 0.1 M H_2SO_4 solution. High-resolution HAADF-STEM images indicate that there are no hydrides formed after long time hydrogen charging. Two types of twin boundaries, i.e., a ∑3 {111} coherent twin boundary (CTB) and a ∑3 {112} incoherent twin boundary (ITB) with 9R structure are observed.

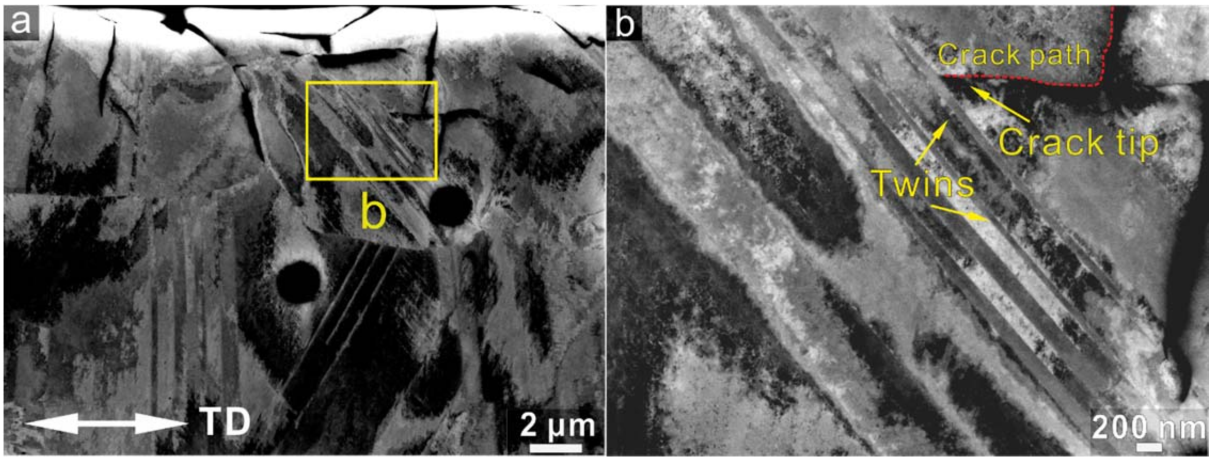

Supplementary Figure 4 | ECC images of cracking after tensile deformation in hydrogen affected regions. The images show that crack propagation can be impeded by twins and twin boundaries. TD refers to the tensile direction.

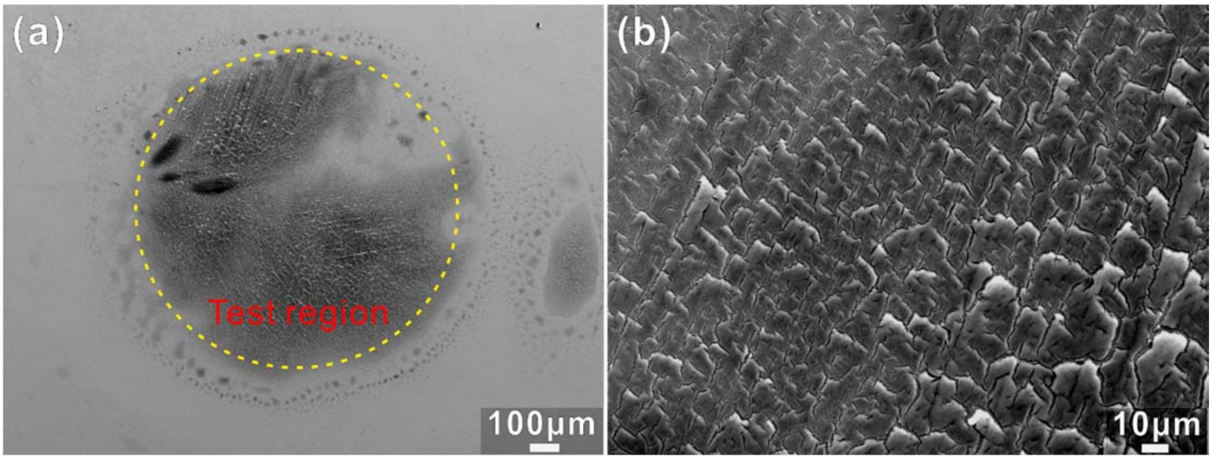

Supplementary Figure 5 | Surface corrosion morphology after potentiodynamic polarization curve testing in a 0.1 M H_2SO_4 solution. The general corrosion feature is related to the fact that the applied potential in the transpassive region exceeded the potential in the passive region, which is similar to that observed in other corrosion resistant alloys.

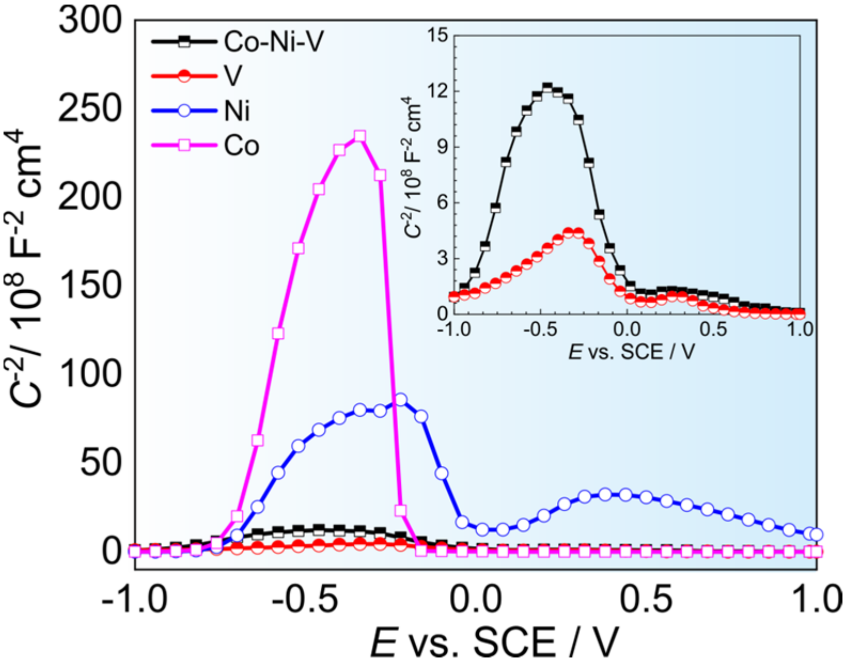

Supplementary Figure 6 | Mott-Schottky plots of the equiatomic CoNiV MEA and pure Co, Ni and V metals in a 0.1 M H_2SO_4 solution.

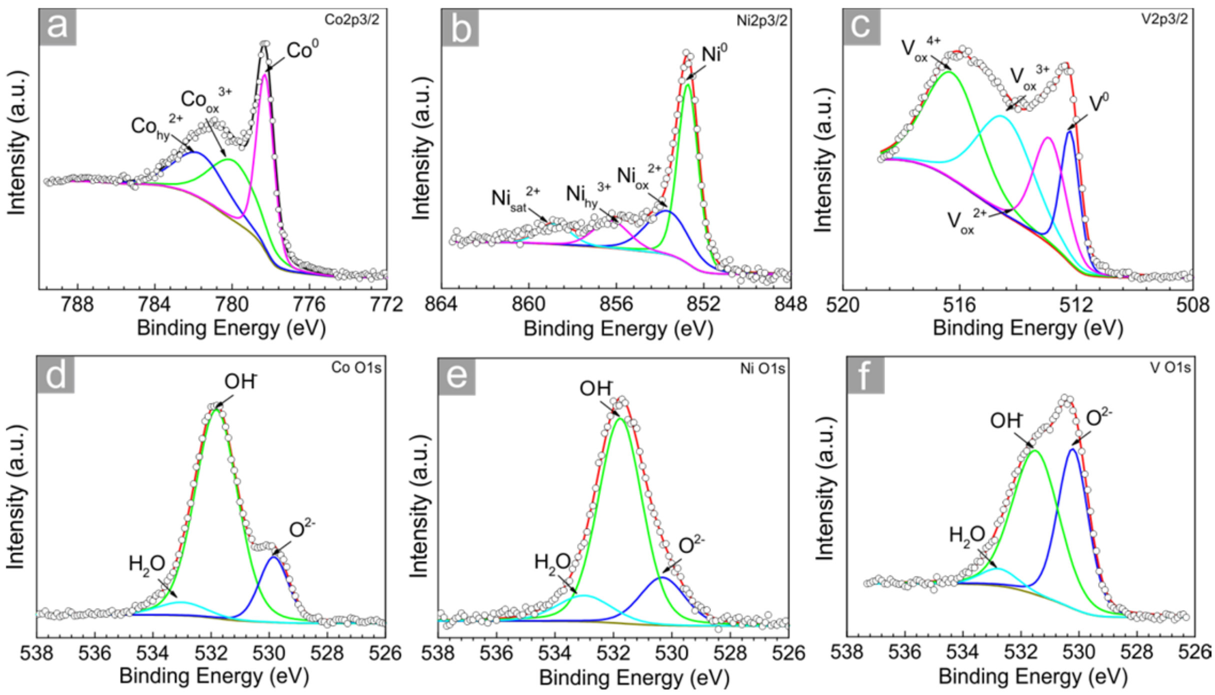

Supplementary Figure 7 | XPS analysis of the composition of the barrier films formed on pure Co, Ni and V elements. a, d Co2*p*3/2 and O1*s*. **b, e** Ni2*p*3/2 and O1*s*. **c, f** V2*p*3/2 and O1*s*.

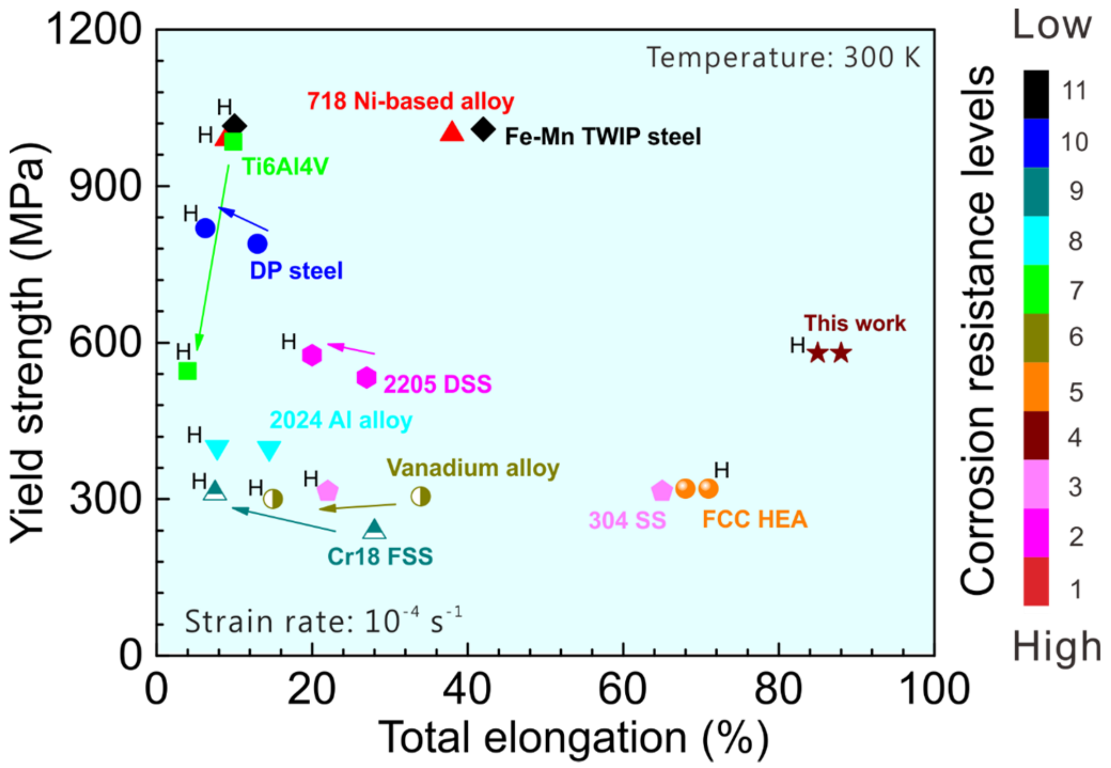

Supplementary Figure 8 | A summary of total elongation vs. yield strength values for various alloys with/without hydrogen. All samples were probed at the same slow strain rate level (10^{-4} s^{-1}) and at the same temperature of 300 K. The arrows in this Ashby diagram show in which direction the yield stresses change when the alloys are exposed to hydrogen.

Supplementary Table 1

Table 1 Chemical composition of the equiatomic CoNiV medium-entropy alloy in weight percent (wt. %).

Elements (wt. %)	C	Co	Ni	V	P+Pb
-	＜0.0001	34.96	34.83	30.22	＜0.0001

Supplementary Table 2

Table 2 Rate of cationic fraction in the barrier films of the equiatomic CoNiV MEA in the test solution.

Elements	V (at.%)				Ni (at.%)				Co (at.%)		
	V^0	$V^{2+}{}_{ox}$	$V^{3+}{}_{ox}$	$V^{4+}{}_{ox}$	Ni^0	$Ni^{2+}{}_{ox}$	$Ni^{3+}{}_{hy}$	$Ni^{2+}{}_{sat}$	Co^0	$Co^{3+}{}_{ox}$	$Co_{hy}{}^{2+}$
Pure metal	14.5	19.8	32.2	33.5	50.8	26.3	13.6	9.3	37.2	34.7	28.1
MEA	10.2	-	29.2	19.3	12.6	5.2	2.5	1.4	9.3	7.4	2.9

Supplementary Table 3

Table 3 Detailed parameters of hydrogen embrittlement (slow strain rate tensile deformation) and corrosion (dilute sulfuric acid solution) for the MEA compared with those observed for other existing metallic materials.

Alloys	Strain rate (s^{-1})	Hydrogen concentration	Ultimate strength No hydrogen (MPa)	Ultimate strength Hydrogen charged (MPa)	Elongation No hydrogen (%)	Elongation Hydrogen charged (%)	Decay (%)	Corrosion current densities (μA cm^{-2})	Corrosion levels
718 Ni-based alloy[1]	1 ×10^{-4}	40 wt.ppm	~1220	~1020	~38	~9	76.3	~0.9	1
Fe-Mn-Al-C TWIP steel [2,3]	1 ×10^{-4}	0.14 wt.ppm	~1000	~1000	~42	~10	76.2	~2000	11
Ferrite/Martensite (F/M) DP steel [4,5]	4×10^{-3}	0.8 wt.ppm	~1168	~1148	~13	~6.3	51.5	~107	10
304 SS [6,7]	8.3×10^{-4}	35 wt.ppm	~720	~650	~65	~22	66.2	~2.5	3
2205 DSS [8,9]	1.3×10^{-4}	5.1 wt.ppm	~772	~800	~27	~20	25.9	~1.0	2
Cr18 FSS [10,11]	1 ×10^{-4}	290 at. ppm	~350	~400	~28	~7.5	73.2	~27	9
V4Cr4Ti alloy [12]	4×10^{-4}	24 wt.ppm	~400	~400	~32	~15	53.1	~7.0	6
Cantor HEA[6,13]	1 ×10^{-4}	8.0 wt.ppm	~545	~571	~68	~71	-4.4	~6.68	5
2024 Al alloy [14]	1 ×10^{-4}	50 wt.ppm	~590	~534	~14.5	~7.8	46.2	~20	8
CoNiV MEA	1 ×10^{-4}	78.2 wt.ppm	~1100	~1090	~88	~85	3.4	~6.53	4
Ti6Al4V [15,16]	1.4×10^{-3}	0.3 wt.ppm	~1180	~1020	~9.9	~4.0	59.6	~10	7

Supplementary References

1 Tarzimoghadam, Z., Ponge, D., Klöwer, J. & Raabe, D. Hydrogen-assisted failure in Ni-based superalloy 718 studied under in situ hydrogen charging: The role of localized deformation in crack propagation. *Acta Mater.* **128**, 365-374 (2017).

2 Kannan, M. B., Raman, R. S., Khoddam, S. & Liyanaarachchi, S. Corrosion behavior of twinning-induced plasticity (TWIP) steel. *Mater. Corros.* **64**, 231-235 (2013).

3 Koyama, M. *et al.* Hydrogen embrittlement associated with strain localization in a precipitation-hardened Fe–Mn–Al–C light weight austenitic steel. *Int. J. Hydrogen Energy* **39**, 4634-4646 (2014).

4 Koyama, M., Tasan, C. C., Akiyama, E., Tsuzaki, K. & Raabe, D. Hydrogen-assisted decohesion and localized plasticity in dual-phase steel. *Acta Mater.* **70**, 174-187 (2014).

5 Fushimi, K. *et al.* Microelectrochemistry of dual-phase steel corroding in 0.1 M sulfuric acid. *Electrochim. Acta* **114**, 83-87 (2013).

6 Luo, H., Li, Z., Mingers, A. M. & Raabe, D. Corrosion behavior of an equiatomic CoCrFeMnNi high-entropy alloy compared with 304 stainless steel in sulfuric acid solution. *Corros. Sci.* **134**, 131-139 (2018).

7 Hatano, M., Fujinami, M., Arai, K., Fujii, H. & Nagumo, M. Hydrogen embrittlement of austenitic stainless steels revealed by deformation microstructures and strain-induced creation of vacancies. *Acta Mater.* **67**, 342-353 (2014).

8 Della Rovere, C., Santos, F., Silva, R., Souza, C. & Kuri, S. Influence of long-term low-temperature aging on the microhardness and corrosion properties of duplex stainless steel. *Corros. Sci.* **68**, 84-90 (2013).

9 Hoyos, J. J. *et al.* In situ synchrotron radiation measurements during axial strain in hydrogen cathodically charged duplex stainless steel SAF 2205. *Mater. Res.* **21**, e20170686 (2018).

10 Potgieter, J., Adams, F., Maledi, N., Van Der Merwe, J. & Olubambi, P. Corrosion resistance of type 444 ferritic stainless steel in acidic chloride media. *J. Chem. Mater. Sci* **2**, 37-48 (2012).

11 Malitckii, E. *et al.* Hydrogen effects on mechanical properties of 18% Cr ferritic stainless steel. *Mater. Sci. Eng., A* **700**, 331-337 (2017).

12 Chen, J. *et al.* Hydrogen embrittlement of a V4Cr4Ti alloy evaluated by different test methods. *J. Nucl. Mater.* **325**, 79-86 (2004).

13 Luo, H., Li, Z. & Raabe, D. Hydrogen enhances strength and ductility of an equiatomic high-entropy alloy. *Sci. Rep.* **7**, 9892 (2017).

14 Zeides, F. & Roman, I. Study of hydrogen embrittlement in aluminium alloy 2024 in the longitudinal direction. *Mater. Sci. Eng., A* **125**, 21-30 (1990).

15 Yuan, B., Yu, H., Li, C. & Sun, D. Effect of hydrogen on fracture behavior of Ti–6Al–4V alloy by in-situ tensile test. *Int. J. Hydrogen Energy* **35**, 1829-1838 (2010).

16 Li, D. G., Long, Y., Liang, P. & Chen, D. R. Effect of micro-particles on cavitation erosion of Ti6Al4V alloy in sulfuric acid solution. *Ultrason. Sonochem.* **36**, 270-276 (2017).
